# A Theoretical Review on the Impact of EFL/ESL Students’ Self-Sabotaging Behaviors on Their Self-Esteem and Academic Engagement

**DOI:** 10.3389/fpsyg.2022.873734

**Published:** 2022-05-04

**Authors:** Fuhua Zhang

**Affiliations:** School of Foreign Language Studies, North China University of Water Resources and Electric Power, Zhengzhou, China

**Keywords:** EFL/ESL student, self-sabotaging behavior, self-esteem, academic engagement, positive psychology

## Abstract

Learner emotions have been considerably emphasized in SLA research and practice with the advent of positive psychology. This has led to a surge of scholarly interest in this strand of research over the past years all around the world. However, the impact of students’ negative emotions such as self-sabotage that actually occur in english as a foreign language (EFL) classrooms on the construction and development of positive learner emotions like self-esteem and academic engagement has been mostly overlooked by second/foreign language researchers. Against this shortcoming, the present review article presented the theoretical and empirical underpinnings of these three crucial variables in SLA focusing on their conceptualizations, dimensions, typologies, related studies, and research gaps. Finally, the study offers a number of practical implications to [EFL/english as a second language (ESL)] teachers, students, teacher trainers, and SLA researchers in order to increase their awareness of learner emotions and the power of such feelings in language teaching and learning processes.

## Introduction

Language learning has long been dismissed as one of the most demanding tasks for teachers and learners due to the intermingled and nested nature of many involving factors, especially those related to emotion and psychology (King and Ng, 2018; MacIntyre et al., 2019; Mercer, 2020). This justifies the centrality of scrutinizing learner emotions and psychological factors in second/foreign language education (Dewaele et al., 2019). To be more precise, along with making efforts to improve positive emotions in learners, english as a foreign language (EFL) teachers are equally expected to hamper and eliminate negative emotions in the class as well (Seligman, 2006). One such negative, damaging learner-related factor is self-sabotage or self-sabotaging behavior(s) that EFL students reveal in their academic career which functions as a negative mechanism bringing about self-destruction (Özçetin and Hiçdurmaz, 2016). It is a personal tendency formed without any external force that determines students’ attitudes and behaviors at school and mobilizes their internal dynamics (Akın, 2012).

Students who use self-sabotaging behaviors, usually postpone or escape the responsibilities, complain and look for excuses, have defensive expectations, are task-oriented, and have poor performance in academia (Özçetin and Hiçdurmaz, 2016). This individual attribute can be temporary or permanent depending on the degree and duration of one’s perceptual conflict with his/her pre-specified goals. However, as research shows, long-term self-sabotage incurs different negative consequences including mental health decline, stress, anxiety, depression, personality disorder, decrease in self-efficacy, satisfaction, internal motivation, and poor performance (Akın et al., 2011; Üzbe, 2013; Özçetin and Hiçdurmaz, 2016). Operationally, students’ self-sabotaging behaviors in this review refers to those damaging behaviors and actions that ruin students’ academic performance and psycho-emotional states. Another area that EFL students’ self-sabotaging behaviors can influence is their self-esteem which is a positive or negative attitude of a person toward him/herself (Burns, 1979). It is an evaluation of self-worth among individuals (Branden, 2001). According to Brown (2007), self-esteem has penetrated into almost all educational aspects to such an extent that no fruitful cognitive or affective activity can be done without considering some degree of self-esteem. The broad scope of the construct of self-esteem has led to a bulk of research on its association with different L2 variables such as language proficiency, motivation, intelligence, language skills, achievement, and many more (Kalanzadeh et al., 2013; Satriani, 2014; Lee et al., 2017, among others). Nevertheless, the impact of student-related psycho-emotional stressors and setbacks (i.e., self-sabotage) on the formation and development of this variable has been limitedly (if any) examined in EFL contexts. It is axiomatic that students’ classroom behaviors play a critical role in shaping their sense of self-worth and attitudes toward their abilities. Consequently, the connection between self-sabotaging behaviors and self-esteem is warranted as these behaviors can reduce or damage self-esteem. In other words, a relationship can be assumed between students’ self-sabotage and self-esteem in that self-sabotaging behaviors like escaping from duties can be the result of a low level of self-esteem or self-worth among the students. This causal association can be rectified by improving students’ self-esteem so that the frequency of self-sabotaging behaviors minimize in academia.

Moreover, the interconnected nature of students’ self-esteem level and their degree of classroom involvement and academic engagement, as endorsed by Virtanen et al. (2016), provided another inspiration for running the current review article. Academic engagement as a dynamic variable in L2 education concerns students’ amount of involvement in classroom activities/tasks that may endure for minutes (Skinner and Pitzer, 2012). It is a realization of motivation on which various personal and contextual factors exert impacts (Guilloteaux, 2016). Of personal factors, research certifies that demographic factors and positive emotions such as resilience, buoyancy, care, interpersonal communication skills, well-being, hope, love, and the like can influence EFL students’ degree of academic engagement (Wilkinson and Kaukko, 2020; Gao, 2021; Zhang, 2021). Despite these valuable scholarly attempts, the relationship between [EFL/english as a second language (ESL)] students’ self-esteem and academic engagement under the influence of a learner-related negative stressor like self-sabotage has been largely ignored in the pertinent literature of this line of research. Students’ self-sabotaging behaviors have a role to play in academic engagement and disengagement of the students as they can influence many educational domains. To put it simply, self-damaging behaviors and practices of students reduce the degree and quality of students’ classroom engagement. Correspondingly, a high level of academic engagement can prevent the occurrence of self-sabotaging behaviors among EFL/ESL students, too. Teachers and administrators in L2 education contexts can use different methods and techniques by which students’ engagement in the process of learning increases, while the likelihood of resorting to negative emotions and behaviors decreases, too.

Therefore, running research on the impact of negative behaviors (e.g., self-sabotage) on learners’ sense of self-esteem and engagement in the class can add to our knowledge of emotions in education. To bridge the gaps and extend the scientific trends in learner psychology, this mini-review article tried to examine the theoretical and empirical background of students’ self-sabotage, self-esteem, and academic engagement and their connection using previous research findings. It also provided clear conceptualizations for each construct and offered practical implications.

## Background

### The Concept of Self-Sabotaging Behaviors

Self-sabotaging behaviors refer to individual actions and decisions that prevent one’s success and attempt to improve his/her performance (Akın, 2012). These behaviors are said to interfere with individuals’ long-term goals and cause various problems in both personal life and academic life (Sertel and Tanriögen, 2019). Such negative behaviors are representations of students’ maladaptive engagement in academia that occur when they get involved in behaviors like procrastination so they can use it as an excuse for their poor performance (Collie et al., 2019). As put by Akın et al. (2011), self-sabotaging behaviors can be divided into two categories of verbal and behavioral self-sabotage. Students with verbal self-sabotaging behaviors strongly state that the cause of their poor performance in academic tasks relates to their external world. They attribute their failure to stress, pain, anxiety, misfortune, and other psychological and physical factors. In contrast, behavioral self-sabotage refers to intentional actions that directly influence one’s performance (Hendrix and Hirt, 2009). It is more active, open, purposeful, and observable by others (Özçetin and Hiçdurmaz, 2016). Students with behavioral self-sabotage intentionally postpone their duties, set difficult goals, avoid practicing, sleep late, use drugs, and so forth (Akın, 2012). Regardless of their type, self-sabotaging behaviors can be transient and situational or chronic and permanent with damaging effects on students’ learning process (Sertel and Tanriögen, 2019).

### The Realizations of Students’ Self-Sabotaging Behaviors

**FIGURE 1:**
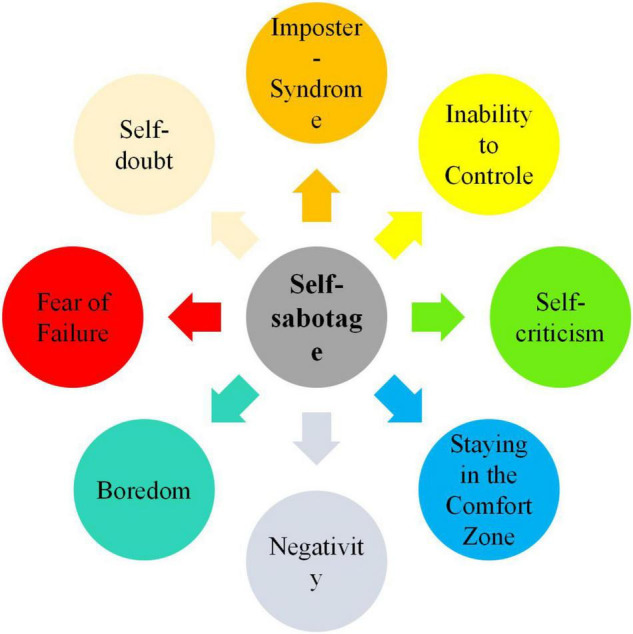
Students’ self-sabotaging behaviors.

There are different behavioral realizations for students’ self-sabotage in the classroom and the learning process as a whole. Some students use procrastination, self-medication *via* drugs, commit self-injuries, and form harmful thought patterns that preclude their learning (Collie et al., 2019; Sertel and Tanriögen, 2019). Other self-destructive behaviors that are the outcome of a conflict between the subconscious mind and rational mind, as illustrated in Figure 1, include: (1) a sense of lack of deserve for success (also called imposter syndrome), (2) feeling of inability to control the situation, (3) overthinking and fearing from failure, (4) self-doubt or low self-efficacy, (5) self-criticism or self-blame for a problem, (6) staying only in one’s comfort zone, (7) negativity, and (8) a sense of boredom (Akın, 2012; Özçetin and Hiçdurmaz, 2016; Sertel and Tanriögen, 2019).

### The Causes and Consequences of Self-Sabotaging Behaviors

Like other learner-related factors that have the psycho-emotional basis, self-sabotaging behaviors can be the result of various issues. As some research studies in this domain indicate, self-sabotage or self-destruction in academic contexts can occur due to students’ sense of ambiguity about success, their past experiences, negative self-perception, maladaptive perfectionism, fear of making mistake, personality, anxiety, task perception and value, pessimism, low self-esteem, low self-efficacy, individual mood, and physical features (Martin et al., 2003; Akın, 2012; Özçetin and Hiçdurmaz, 2016; Sertel and Tanriögen, 2019). In addition to these personal factors, students’ use and resort to self-sabotaging behaviors can be the outcome of contextual and organizational factors such as classroom climate, classroom culture, and organizational structure as well (Sertel and Tanriögen, 2019). Moreover, teachers’ pedagogical behaviors and practices in the class are by no means irrelevant to the development of this aversive emotion in EFL students (i.e., self-sabotage).

If these damaging behaviors are not treated in academia, they can weaken or even destroy students’ mental and physical health, motivation, sense of hope, enjoyment, satisfaction, harmony, happiness, and psychological well-being (Özçetin and Hiçdurmaz, 2016). Self-sabotage can also lead to one’s emotional dissatisfaction, anxiety, depersonalization (also called self-alienation), depression, social isolation, burnout, and low academic performance (Akın, 2012). Additionally, these negative behaviors can directly influence many other learner-related factors like self-esteem and classroom engagement that have been overlooked by researchers in EFL/ESL contexts.

### The Concept of Self-Esteem: Typologies and Cognates

The concept of self-esteem, as one of the most widely explored psychological variables in education, is defined as how much worth or value a person considers for him/herself as an individual (Rosenberg, 1989; Harter, 1999; Morin and Racy, 2021). Musitu et al. (1988) dismissed self-esteem as a worth and evaluative quality of the cognitions and behaviors manifested in the amount of personal satisfaction. It is divided into three types according to level, namely *inflated*, *high*, and *low* self-esteem (Baumeister and Boden, 1998; Piff, 2014). Individuals with inflated self-esteem consider themselves better than others all the time and try to underestimate others’ abilities. However, those with high self-esteem are prone to love and accept themselves by trusting in their own abilities. In contrast, people with a low self-esteem level do not believe in themselves and their abilities to carry out a task. Hence, their performance is not good and feel a lot of anxiety and pressure. Moreover, Rosenberg et al. (1995) classified self-esteem into *global* and *specific* according to the degree of coverage. Global self-esteem is a general sense of self-worth in various domains, while specific self-esteem is present in only a certain aspect of one’s life or career.

In the existing literature, different cognate terms have been proposed and used instead of or synonymous with self-esteem including self-concept, self-efficacy, self-competence, self-worth, and self-confidence. Although they seem similar, they have different concerns and denotations. Self-concept is a person’s overall self-image of him/herself and his/her abilities (Jordan, 2020), while self-efficacy is one’s confidence is successfully accomplishing a task (Bandura, 1997). Self-competence concerns one’s perceptions of ability in broad academic domains (Harter, 1982), while self-worth is simply self-love or one’s favorable opinion of him/herself (Bogee, 1998).

### Self-Esteem and SLA Research

Self-esteem is a psychological variable that has witnessed an abundance of research in educational psychology and other fields. It is of paramount importance in SLA due to the unique nature of L2 education (Guban-Caisido, 2020). After positioning itself in the body of knowledge concerning the interaction between psycho-emotional factors and SLA, many studies were conducted on the correlation between self-esteem, positive emotions, academic motivation, anxiety, achievement, persistence, flexibility, and the like (Leary and MacDonald, 2003; Dewaele, 2011; Hisken, 2011; Koosha et al., 2011; Basco and Han, 2016; Moriya, 2019). Moreover, SLA researchers, in the past decade, have taken another step and examined the impact of students’ self-esteem on their language proficiency and performance in different language skills such as speaking (Mandokhail et al., 2018; Satriani, 2019), reading comprehension (Piran, 2014; Stranovska and Gadusova, 2020), writing (Fahim and Rad, 2012), and listening skills (Hayati and Ostadian, 2008; Itzchakov and Weinstein, 2021). Although these studies have made the concept more tangible and vivid, they have been mostly focusing on the effect of positive emotional variables on self-esteem or the role of this concept in students’ language competency domains. The missing part is the impact of or relationship between students’ negative emotions and classroom actual behaviors such as self-sabotage on the development and operation of students’ self-esteem in EFL/ESL contexts. Hence, the present review article was set to deal with these gaps in the literature and examine self-esteem along with self-sabotage and academic engagement as they have mutual impacts on each other like many other psycho-emotional variables in SLA.

### The Definition of Student Engagement

With the rise of educational psychology and positive psychology, teachers and practitioners paid special attention to students’ classroom engagement as a vital element to produce academic success in various aspects (Gu and Sun, 2021; Zhang, 2021). As simply put by Skinner and Pitzer (2012), the notion of student engagement has to do with students’ amount and duration of classroom involvement in the given activities. It is a highly desired objective in language learning that facilitates the ground for several human competencies (Sinatra et al., 2015).

Moreover, engagement is a direct sign of motivation in students that provides energy and reason for academic investment and success (Phillips, 2015). Additionally, as maintained by Guilloteaux (2016), engagement as a dynamic construct can be affected by different factors internal or external to the person yet it shapes learners learning enthusiasm, commitment, involvement, hard-work, and determination.

### The Dimensions and Influencing Factors of Student Engagement

**FIGURE 2:**
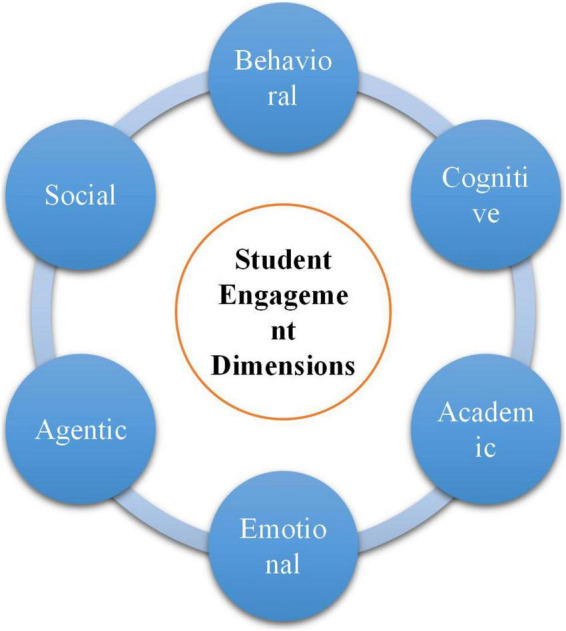
The dimensions of student engagement.

According to DeVito (2016), the construct of student engagement has a multi-dimensional essence including behavioral, emotional, cognitive, agentic, academic, motivational, and social dimensions. They have part-to-part, part-to-whole, and whole-to-part interactions to produce the overall engagement in the classroom (Symonds et al., 2021). In similar research, Reschly and Christenson (2012) explained the dimensions of engagement (Figure 2) as what follows:

➢ ***Behavioral engagement:*** has to do with pupils’ conformity and active classroom participation extracted *via* tasks/activities.
➢ ***Cognitive engagement:*** concerns students’ psychological investments during their learning process by means of complex strategies that they employ during completing a task.
➢ ***Emotional engagement:*** is associated to students’ internal emotions and states together with their reactions to their learning process.
➢ ***Academic engagement:*** pertains to various psychological and behavioral efforts that students make to master the desired knowledge and skills.
➢ ***Agentic engagement:*** has to do with the degree of students’ influence and role in increasing the quality of both learning and teaching processes.
➢ ***Social engagement:*** as the name suggests, deals with learners’ deep involvement in social-oriented tasks and activities that are aimed at stimulating their problem-solving abilities and social interaction skills.

It is essential to mention that all these dimensions/components have an interactive interplay to ultimately lead to students’ classroom engagement. In addition to the dynamism and interactional nature of the construct, students’ degree and duration of academic engagement in a class might be affected by different psycho-emotional experiences and events during an activity (Eccles, 2016). In this regard, Guilloteaux (2016) categorized factors that affect student engagement into *phenomenological*, *individual-demographic*, and *instructional factors*. Given these factors and the complex nature of engagement, it can be contended that EFL/ESL students’ self-sabotaging behaviors and self-esteem that belong to the second category of influential factors can influence their academic engagement, as well.

### Correlates of Student Engagement

Student engagement as a positive variable flourished by PP proponents has been scrutinized in different contexts. Scientific findings have pinpointed that it is positively correlated with effective learning, persistence, retention, motivation, resilience, ambiguity tolerance, agency, willingness to communicate (WTC), and learning perception (Wimpenny and Savin-Baden, 2013; Radmehr and Karami, 2019; Dao and Sato, 2021; Gu and Sun, 2021; Hiver et al., 2021; Wind, 2021; Zhang, 2021). Other than learning benefits, student engagement can meaningfully contribute to students’ socialization, life satisfaction, and psychological well-being (Trowler and Trowler, 2010; Zepke, 2015). Furthermore, it can be argued that student engagement has a potential direct association with many positive emotions introduced by PP such as buoyancy, stroke, care, hope, joy, passion, interpersonal communication skills, sense of closeness, connectedness, and many more that entail further research worldwide.

## Final Remarks

In this study, it was maintained that students’ emotions play a significant role in L2 education deserving practitioners’ prime attention. It was also argued that EFL/ESL students’ negative emotions such as self-sabotage in the classroom influence various aspects of their language learning. Two such areas were substantiated to be students’ self-esteem and academic engagement based on scientific findings of the literature. In light of these outcomes, it is contended that this review article can be of valuable implications for EFL/ESL teachers, students, teacher trainers, and researchers interested in positive psychology and educational psychology. EFL/ESL teachers may find this study useful in that they can realize the criticality of taking students’ emotions into consideration to make L2 education as enjoyable as possible. They can also use the study to think deeply about students’ self-sabotaging behaviors in the classroom and get ready to deal with such challenges that prevent learning. Likewise, students can benefit from this article in that identify the importance and degree of impact that their emotions and classroom behaviors have in their academic success, engagement, and sense of self-esteem. Moreover, teacher trainers may use this study to plan, devise, and offer effective training programs for EFL/ESL teachers focusing specifically on how to locate, prevent, and turn negative student emotions like self-sabotage to positive outcomes. They can provide teachers with useful techniques and strategies to deal with these setbacks and improve students’ academic engagement and achievement. Likewise, this review article might be useful for researchers in that they can get some fresh ideas about researching student emotions in SLA and the existing gaps in this domain. For instance, as reviewed, most of the studies are one-shot, correlational studies that provide an incomplete image of psycho-emotional factors of SLA. Hence, future studies are suggested to use qualitative and mixed-methods research designs. The effect of treatment and training courses on preventing EFL/ESL students’ self-sabotaging behaviors is also a fresh idea to be empirically explored. The variables examined in this article are influenced by cultural variation, hence cross-cultural studies are highly suggested to L2 researchers. Finally, the association between students’ self-sabotaging behaviors and other constructs common in PP such as resilience, buoyancy, immediacy, interpersonal communication, agency, self-regulation, emotioncy, strategic investment, autonomy, growth mindset, motivational intensity etc. is also recommended to enthusiastic L2 scholars.

## Ethics Statement

The studies involving human participants were reviewed and approved by North China University of Water Resources and Electric Power Academic Ethics Committee. The patients/participants provided their written informed consent to participate in this study.

## Author Contributions

The author confirms being the sole contributor of this work and has approved it for publication.

## Conflict of Interest

The author declares that the research was conducted in the absence of any commercial or financial relationships that could be construed as a potential conflict of interest.

## Publisher’s Note

All claims expressed in this article are solely those of the authors and do not necessarily represent those of their affiliated organizations, or those of the publisher, the editors and the reviewers. Any product that may be evaluated in this article, or claim that may be made by its manufacturer, is not guaranteed or endorsed by the publisher.
